# The stressful tumour environment drives plasticity of cell migration programmes, contributing to metastasis

**DOI:** 10.1002/path.5395

**Published:** 2020-03-14

**Authors:** Savvas Nikolaou, Laura M Machesky

**Affiliations:** ^1^ Division of Cancer Metastasis and Recurrence CRUK Beatson Institute Glasgow UK; ^2^ Institute of Cancer Sciences University of Glasgow Glasgow UK

**Keywords:** cancer, invasion, metastasis, collective cell migration, Rho GTPase, Rac1, RhoA, Cdc42, microenvironment, macropinocytosis, chemotaxis, durotaxis

## Abstract

Tumours evolve to cope with environmental stresses or challenges such as nutrient starvation, depletion of survival factors, and unbalanced mechanical forces. The uncontrolled growth and aberrant deregulation of core cell homeostatic pathways induced by genetic mutations create an environment of stress. Here, we explore how the adaptations of tumours to the changing environment can drive changes in the motility machinery of cells, affecting migration, invasion, and metastasis. Tumour cells can invade individually or collectively, or they can be extruded out of the surrounding epithelium. These mechanisms are thought to be modifications of normal processes occurring during development or tissue repair. Therefore, tumours may activate these pathways in response to environmental stresses, enabling them to survive in hostile environments and spread to distant sites. © 2020 The Authors. *The Journal of Pathology* published by John Wiley & Sons Ltd on behalf of Pathological Society of Great Britain and Ireland.

## Introduction

Metastasis remains a mysterious and untreatable feature of many cancers. If a primary tumour is detected at early stage, it can often be removed by surgery and the patient will have a high possibility of survival. However, failed early detection of the primary tumour can result in cancer metastasis, which is responsible for more than 90% of cancer‐related deaths. This is primarily because of the complicated nature of tumour dissemination and its resistance to various therapies 1. It is not well understood how tumours acquire the ability to metastasise to distant sites. Here, we explore the idea that a driver of metastasis may be provided by the hostile microenvironment triggering stress responses that lead to hypervariability of normal programmes regulating cell migration, nutrient uptake, and tissue organisation.

Evolution of tumours can be compared with evolution of organisms, as selection takes place in tumours and more successful mutations are preserved over time, similar to Darwin's notion of ‘survival of the fittest’. An interesting idea was put forth by Kirschner and Gerhart in their book *The Plausibility of Life* 2, proposing that each system in a cell or an organism has evolved to contain built‐in modules of variability, which can be activated under stress or in the face of new opportunities. This variability could confer survival advantages and allow relatively large programme changes to occur in a flexible way under stress. If we think of a tumour not as a randomly changing ball of cells, but rather as an emerging system undergoing selection to survive in our bodies by hijacking and combining our intrinsic variability programmes, we can think differently about what lies behind complex phenomena such as metastasis of cancer. Multiple cell processes could be subject to this selection and hypervariability activation under stress, but here we focus on tissue organisation, cell migration, and nutrient uptake via macropinocytosis, which uses the cell migration machinery.

Cell migration is a fundamental process in multicellular organisms and is characterised by a coordinated movement of individual or multiple cells in a specific direction and location. Embryo formation, wound healing, and immune system function require cell migration in multicellular organisms, highlighting its fundamental importance for life. Pathways driving migration can be turned on/off depending on the circumstances, but adult epithelia, from which many solid tumours or carcinomas arise, show limited migration and tend to remain attached to neighbouring cells in an organised way.

**Figure 1:**
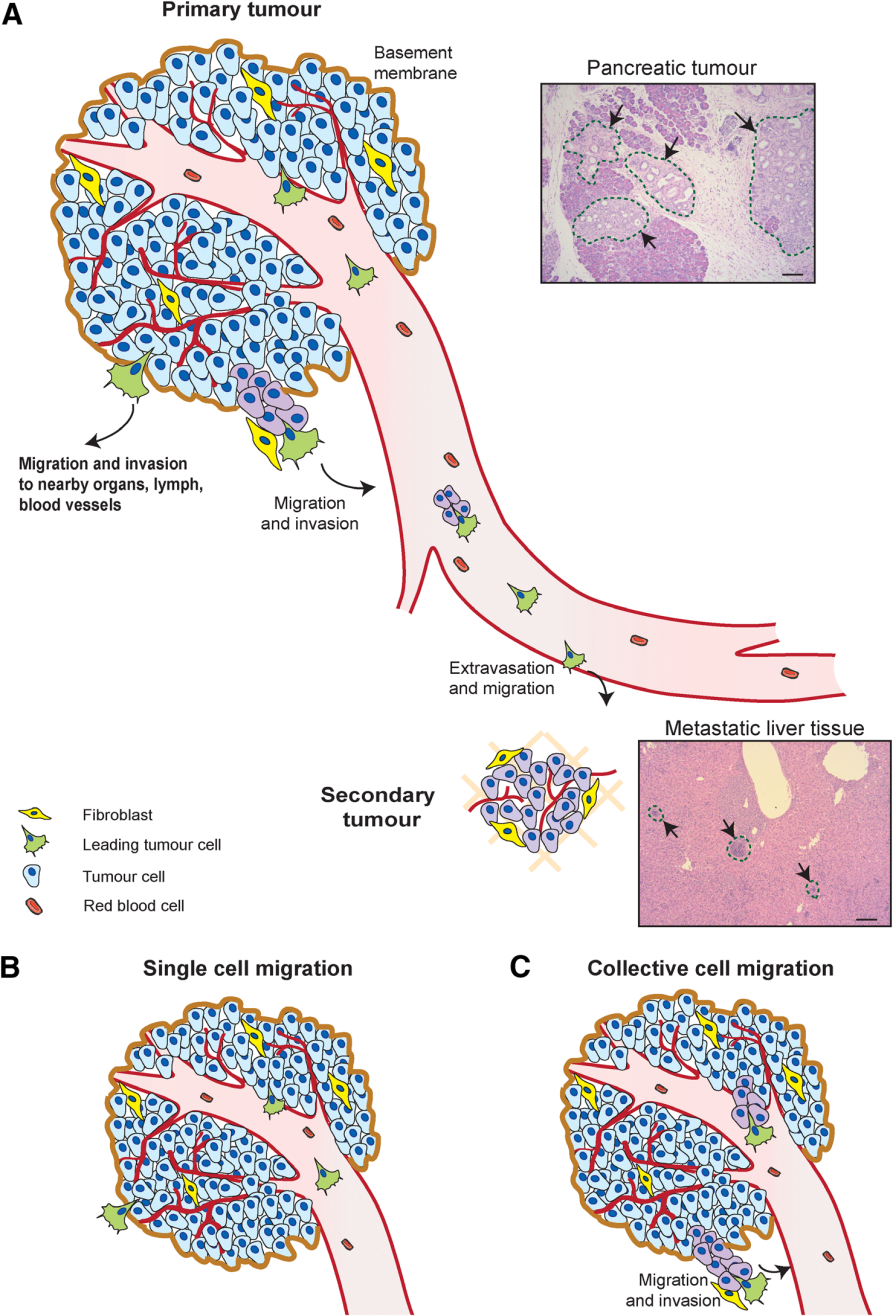
Cell migration and metastasis. (A) Illustration of the process of metastasis from a primary tumour site towards a secondary site, here shown as the liver. Cancer cells have the ability to acquire a motile phenotype and migrate (either in clusters or as individuals). Fibroblasts can assist the migration and invasive process and even serve as leader cells in the invasion 3, 4. Tumour cells invade through basement membranes and extracellular matrix and enter the bloodstream via intravasation. The cancer cells can travel through the bloodstream and extravasate at a secondary site where they will form new tumours (metastasis). Representative haematoxylin and eosin (H&E)‐stained sections taken from mouse Pdx1::Cre; Kras^G12D^; P53^R172H^ pancreatic tumour and liver tissue with metastasis. Scale bars: 100 μm. (B, C) Two examples of cell migration [single cell (B) and collective (C)] which will allow cancer cells to invade and metastasise to a distant site through either the bloodstream or lymph system, or by invading through the extracellular matrix to a nearby organ.

Metastasis is a complex multi‐step process whereby cancer cells gain motility to cross biological barriers and colonise distant sites. This process initiates with cancer cells escaping the primary tumour site, sometimes directly through tumour blood vessel vascular basement membranes or alternatively through epithelial basement membranes and extracellular matrix (ECM) to home to and enter blood vessels through a process called intravasation (Figure 1A). Cells can also enter the lymph system and be carried to a distant organ or migrate along tissue highways such as lymph vessels, nerves, and interstitial spaces, where they will eventually reach a new site (Figure 1A). Although only a few cells might survive in the foreign environment, they may form micrometastases, which can lie dormant or be suppressed by the host immune system until they later grow into metastases 5.

## From epithelia to single cells – actin drives individual migration

Epithelia are composed of sheets and tubes of cells with apical and basal polarity and have both a specific identity and a niche where they reside relative to other cells and the extracellular matrix. The regulation of epithelial homeostasis has been extensively described in the literature (reviewed in 6, 7). Epithelial cells in adult tissues were thought to be largely static, but are now known to migrate during turnover in the normal intestine 8 and in wound repair. Oncogenes and tumour suppressors can initiate transformation of epithelial cells and cause them to deviate from their normal programmes within the tissue. Following transformation, epithelial cells gradually lose characteristics of their epithelial identity and gain the ability to access distant sites in the body by migrating away from their neighbours and their niche.

Cells can escape tumours either as single entities or as collectives (Figure 1). Single cells generate protrusions, modulate their adhesion, and use their actomyosin cytoskeleton to generate forces and propel through barriers and across the ECM (Figure 1B). Typically, cells establish a front–rear polarity by positive and negative feedback mechanisms that create dominant centres of actin assembly, adhesion or contractile force generation. The Rho GTPase family of proteins plays key roles in regulating single cell migration by orchestrating activation and dynamics of multiple actin cytoskeletal regulating proteins. While the networks of regulation are complex, it is typically thought that Rac1 mainly regulates actin‐based protrusions; RhoA primarily mediates actomyosin contractility; and Cdc42 coordinates polarity of adhesion, contractility, and cell protrusion (reviewed in 9).

Individual cell migration is controlled by the Rho GTPase Rac1 interacting with the Scar/WAVE complex, resulting in Arp2/3 activation and explosive nucleation of branched actin filaments (reviewed in 10). As a result of multi‐valent binding interactions, centres of actin nucleation cluster together with receptors and associated signalling scaffold proteins and form domains resembling phase separations 11, 12. These clusters allow sustained regional actin polymerisation and may trigger crosstalk with other signalling molecules, which enables cells to push the plasma membrane outward, forming protrusions such as lamellipodia, filopodia, and invadopodia. Lamellipodia are sheet‐like protrusions containing dendritic branched actin networks formed by the activity of Rac1 and Scar/WAVE. Rac1 binds directly to two different sites on the Scar/WAVE complex, which may have distinct roles during activation and function of the complex as well as being involved in complex multimerisation 13, 14. In addition, we have recently described a new negative regulator of the Scar/WAVE complex, CYRI. CYRI can compete with the Scar/WAVE complex for binding to active Rac1 15, 16, but rather than preventing activation of Scar/WAVE, CYRI raised the threshold of Rac1 activation at which the cell is capable of making a burst of actin assembly. Actin‐based lamellipodia are also key structures used in uptake of extracellular particles such as pathogens. Loss of CYRI led to an increase in uptake of salmonella in mice, implicating Rac1 and CYRI in actin‐mediated engulfment 17. CYRI thus likely raises the threshold at which activation of Rac1 leads to actin assembly by the Scar/WAVE complex to assemble multiple types of actin structure, including phagocytic cups for pathogen uptake.

**Figure 2:**
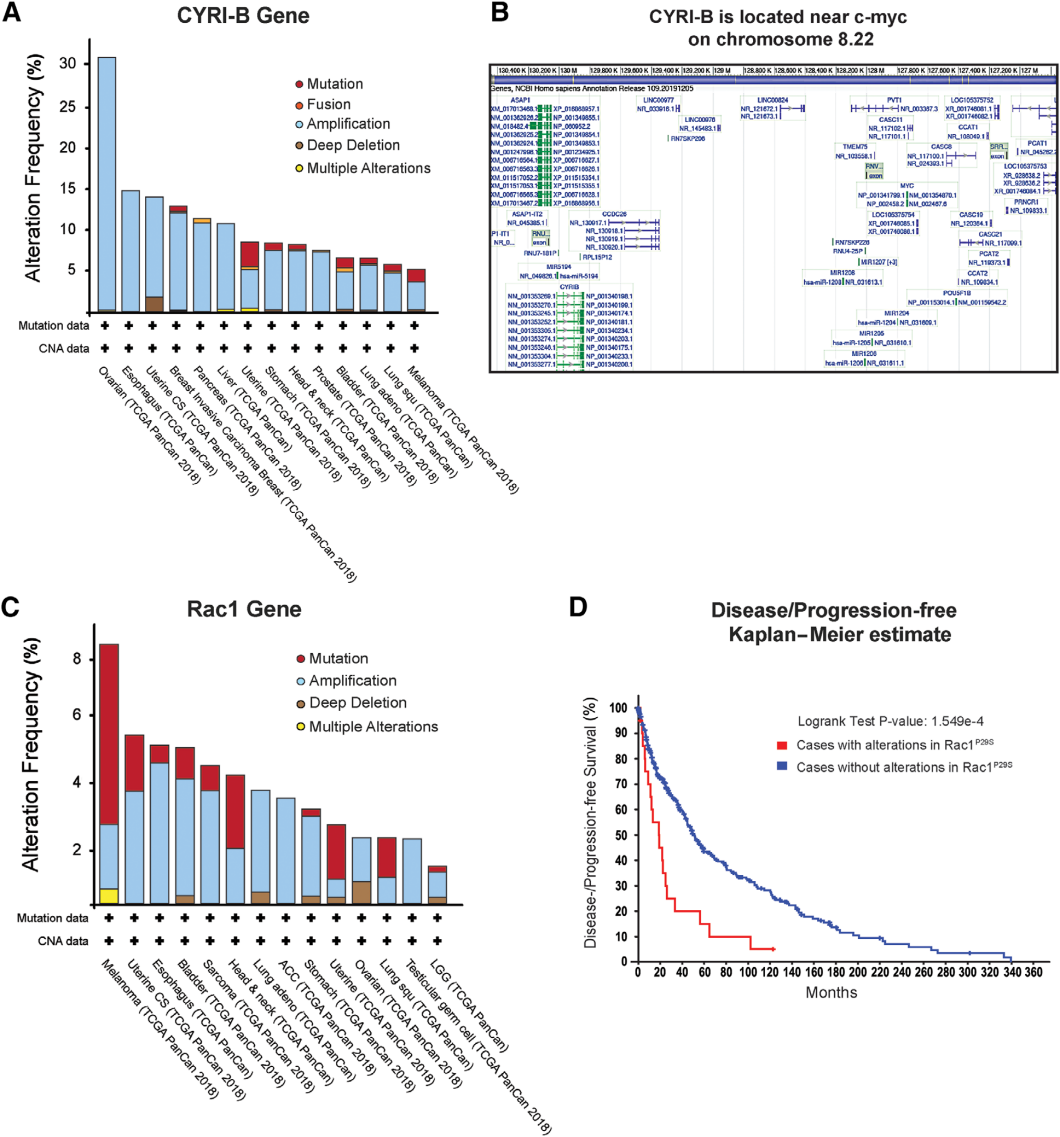
CYRI‐B and Rac1 can be altered in different types of cancer. (A) The alteration frequency of CYRI‐B in different types of cancer. (B) The CYRI‐B gene is located near *MYC* on chromosome 8q 22. From NCBI Human Genome resource https://www.ncbi.nlm.nih.gov/genome/guide/human/. (C) Alteration frequency of Rac1 in different types of cancer. (C) Kaplan–Meier survival curve of patients with or without Rac1^P29S^ somatic mutation. Data obtained from cBioPortal for Cancer genomics database 18, 19.

As a driver of motility and cell cycle progression, the Rac1–Scar/WAVE pathway is linked to cancer progression and metastasis in many tumour types. CYRI‐B is overexpressed in many cancers, but the significance of this, if any, is unknown (Figure 2A). CYRI‐B lies near the gene encoding c‐Myc on human chromosome 8 region q22 (Figure 2B), so it may be a passenger amplification along with *MYC* in cancer. Rac1 is overexpressed either by gene amplification or by mRNA upregulation (Figure 2C), usually correlating with poor patient survival (Figure 2D). In melanoma, Rac1 is frequently mutated at proline 29 (P29S), which raises its activation levels by increasing the GDP/GTP nucleotide exchange rates 20, 21. This point mutation promotes actin assembly and lamellipodia formation and it was also shown to increase proliferation independently of the ERK/MAPK pathway in response to growth factor deprivation 22. Additionally, Rac1 P29S can drive a mesenchymal transcriptional switch via serum response factor 23. This is clinically interesting, as BRAF kinase is one of the most frequently mutated oncogenes in melanoma and this mesenchymal switch promotes both tumourigenesis and resistance to BRAF kinase inhibitors 24. TCGA data from 366 patients show that 6% have mutated Rac1, with a significant effect on disease/progression‐free survival (Figure 2C). Melanoma patients with Rac1^P29S^ somatic mutation are resistant to RAF inhibitors such as vemurafenib and dabrafenib, whereas silencing of Rac1 in these cells reversed this resistant phenotype 24. Thus, this mutation may be selected during chemotherapy treatment, contributing to cancer resistance and relapse. A new meta‐analysis of 14 different studies with a total of 1793 cancer patients found that high levels of Rac1 in different cancer types including prostate, breast, hepatocellular, and non‐small‐cell lung cancer were linked to tumour malignancy, indicating that Rac1 overexpression may be a potential biomarker for cancer progression 25.

Cells have multiple machineries that contribute to migration and these act together depending on the pericellular microenvironment. In the absence of protrusions, e.g. by deletion of the Scar/WAVE or Arp2/3 complexes, cells can still polarise and move, albeit much less efficiently 26, 27. Additionally, protrusive forces are not effective in conditions of very low adhesion or in extreme confinement 28. The contractile machinery of cells can drive motility in most of these cases, sometimes acting as a piston to generate force against the walls of the confined space or to squeeze through tight spaces 29. Contractile activity is largely coordinated by RhoA, which regulates the activity of the major myosin activating pathways such as Rho‐kinase and myosin light chain kinase, as well as inhibiting the myosin inactivating phosphatase. Rho‐mediated contractility is a major driver of tumour matrix remodelling and motility of cancer cells 30, 31.

In addition to protrusion and contractile squeezing, adhesion forces have a strong effect on how cells migrate and whether they can efficiently move in different environments. Adhesion and its role in migration have been well studied and some informative reviews can be found 32, 33, 34, 35, 36. To summarise briefly, new adhesions form in the leading edge of lamellipodia when cells migrate across a rigid substrate. Nascent adhesions mature as actin assembles and flows retrogradely towards the cell body and is bundled into stress fibres and actin cables. Many focal adhesion proteins are tension‐sensitive and respond to increased force 36. In softer environments and in 3D matrix, the process is similar, but focal adhesions tend to be smaller and form in regions where the cell contacts matrix fibres 37. As focal adhesions have differing compositions and sub‐types, there is still a lot to learn about how they respond to force and the role of individual protein components in signalling and mechanics.

Actin not only drives protrusion at the plasma membrane but also plays a key role in sculpting membranes that drive endocytic uptake, trafficking, and recycling. Recycling of receptors such as integrins and receptor tyrosine kinases (RTKs) regulates adhesion to the ECM as well as actin organisation for migration towards nutrient gradients 38. The Scar homologue and Arp2/3 complex activator, WASH, complex facilitates the recycling of specific proteins from the plasma membrane to the leading edge of invading cells 38. This mechanism is essential in recycling of proteins involved in invasion and metastasis, including various integrins, membrane spanning metalloproteases such as MT1‐MMP, and RTKs such as EGFR 39, 40. A recent study also showed that upregulation of proteins involved in recycling such as clathrin light chain b (CLCb) and dynamin‐1 (Dyn1) can be correlated with poor prognosis in patients with non‐small‐cell lung cancer 41. These two proteins regulate the recycling of EGFR through clathrin‐mediated endocytosis, and upregulation of both CLCb and Dyn1 can enhance EGFR recycling to plasma membrane, leading to increased invasion and metastasis *in vivo* 41. Thus, individual cell migration is driven by coordination of protrusion, contractile function, adhesion, and trafficking. All of these processes depend on actin dynamics and are controlled by signals from membrane receptors and contact with the matrix.

## Collective cell migration

During embryogenesis, tissue and tube formation is achieved through collective cell migration. Collective cell migration also occurs in the adult intestine, a tissue that turns over rapidly and proliferates mostly near the base of the finger‐like villi in structures called crypts. It has long been the subject of debate whether intestinal epithelial cells actively migrate using protrusions or are just passively pushed along as they crowd due to proliferation. New evidence shows that intestinal epithelial cells in mice use active migration, with Arp2/3 and actin‐based protrusions against the basement membrane 8. This is a nice example of collective migration occurring in normal adult tissue as a part of regeneration. Many types of tumours also display prominent collective invasion. Histological 3D reconstruction of several tumour types revealed that most invasion near a tumour is collective and often finger‐like 42. This type of invasion is also linked to poor prognosis 43, 44. Whether collective migration of tumour cells results from a wound‐healing response, an aberrant developmental programme, or some combination remains to be understood.

When cells migrate as a collective, they display similar molecular mechanisms to single cell migration, such as protrusion, adhesion, and contractile forces, but they also require coordination of cell–cell adhesion or repulsion. Individual cells dynamically alter their actin cytoskeleton to establish a front–rear polarity axis. Conversely, during collective cell migration, the cluster of cells will establish a polarised axis across several cell lengths, by having leader cells at the front and follower cells at the back (Figure 1C). The two types of cells present in the cluster exhibit differences in signalling, gene expression, and morphology. Indeed, leader cells have a more migratory and protrusive phenotype and thus are thought to direct orientation of movement and the speed of the cluster in response to environmental stimuli 45.

**Figure 3:**
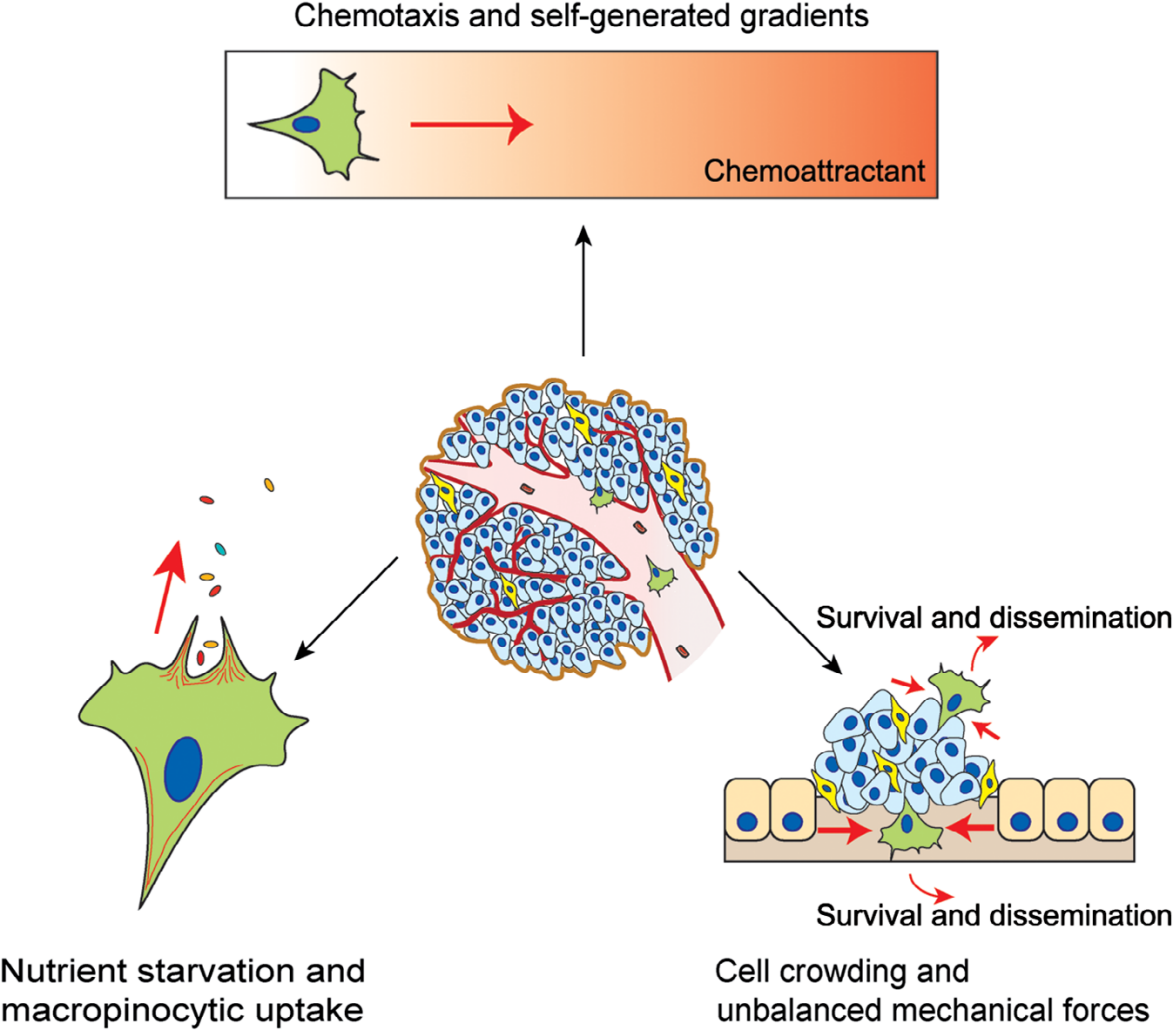
Pressures driving cell migration. Stresses and imbalances associated with the tumour environment can promote cell migration and can lead to metastasis. Common drivers include imbalances in attractants due to consumption by the primary tumour, leading to chemotaxis; nutrient starvation leading to migration and macropinocytic uptake; cell crowding and unbalanced mechanical forces, leading to extrusion, invasion, and migration.

**Figure 4:**
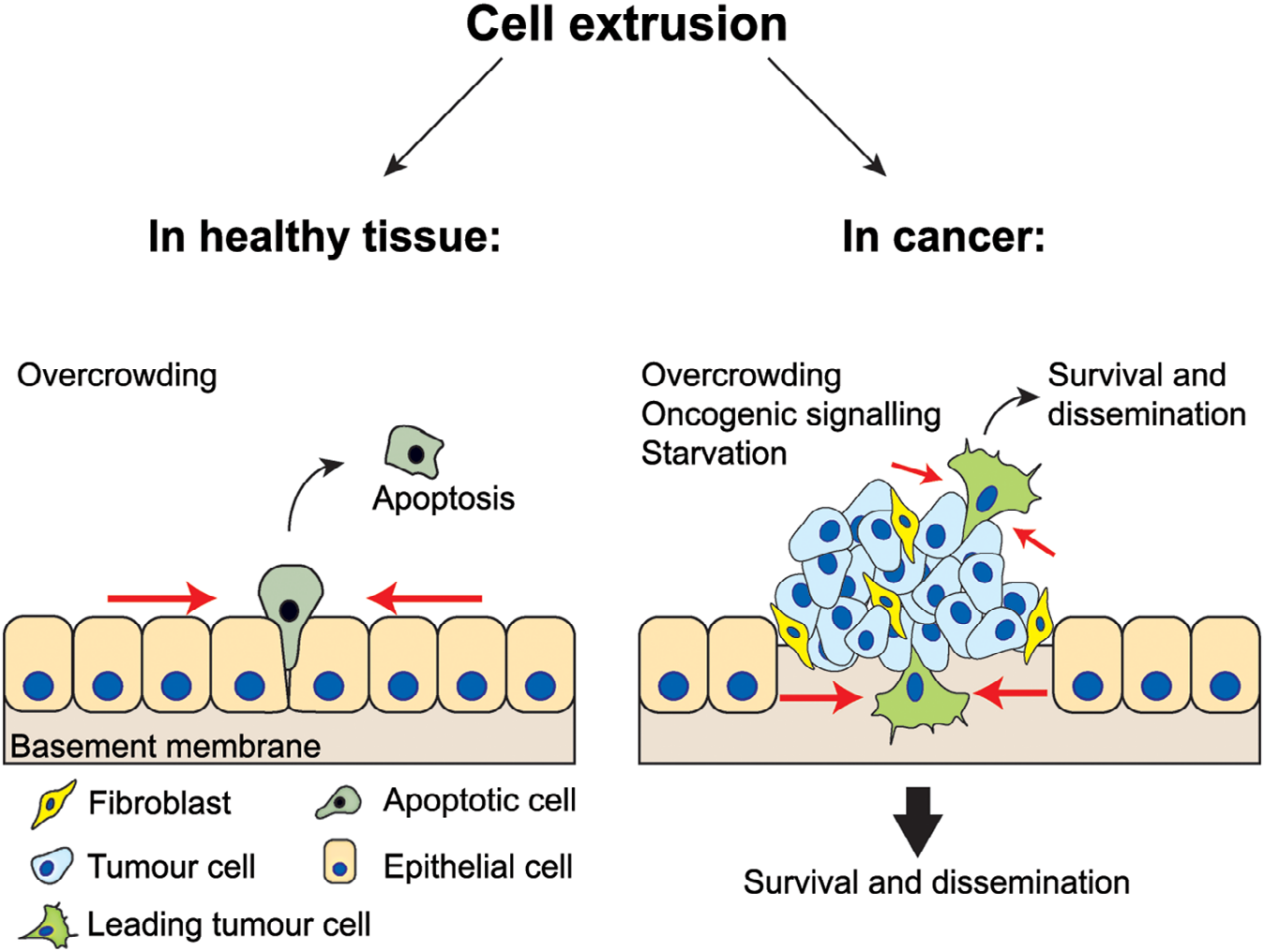
Cell extrusion in health and disease. In healthy tissue, cells can be extruded apically from the epithelial monolayer, due to overcrowding, and undergo apoptosis. In tumours, cancer cells can be extruded (due to overcrowding, oncogenic signalling, and/or starvation) either apically or basally towards the basement membrane and invade through the extracellular matrix to gain access to interstitial spaces and distant organs.

For collective cell migration to be successful, sufficient coordination of cell–cell and cell–matrix interactions is required. Therefore, understanding the role of leader/follower cells within an adherent group during collective migration is critical. Studies from model organisms such as fish, flies, and mammals suggest that cells at the edges of a cluster sense microenvironmental cues, allowing them to become leader cells 46, 47, 48. Alternatively, clusters may contain cells predisposed to become leader cells, which migrate towards the front, where they orchestrate and direct collective migration 49, 50. A third possibility is that a different type of cell, such as a fibroblast, could take over as a leader of collective migration – which can lead to cancer invasion (Figure 1C and 3, 4). A new study using a 3D microfluidic device that enables real‐time imaging of mammalian cell clusters during migration characterised the dynamics of leader and follower cells and found that pre‐existing keratin 14 (K14)‐positive cells in a tumour organoid have the potential to become leader cells. These cells can polarise and migrate as leaders, due to environmental stimuli from the tumour microenvironment as well as chemokine gradients, which are sensed by the fibrillar collagen receptor DDR2 and the DF1 chemokine receptor CXCR4, respectively 51.

Leader and follower cells have different roles in collective migration during normal embryonic development events, such as in angiogenic sprouting or neural crest migration. Single‐cell RNA sequencing of invading neural crest collectives revealed that leader cells have upregulation of genes essential in signalling and motility. Examples include Rho‐GTPases, actin cytoskeletal genes and Wnt/PCP (planar cell polarity) genes 52. However, even within the neural crest, the trunk versus cranial neural crest cells use different mechanisms for collective migration; cranial migration of chick and zebrafish showed no continuous presence of a leader, whereas trunk migration showed an established leader directing the follower cells 53. The Wnt/PCP pathway establishes proper polarisation of cells through an asymmetric distribution of members of this pathway in an individual cell by antagonising other protein complexes intracellularly 54, 55. This asymmetry can then be distributed to the adjacent cells through intercellular protein–protein interactions and therefore generate a local polarity within the cluster enabling a proper coordinated migration 55, 56. Wnt/PCP pathways are frequently hijacked in cancers and lead to aberrant tissue organisation, inappropriate identity, and stemness features 55, 56.

Recently, the importance of energy regulation was highlighted for collective migration and leader/follower status of cells. Leader cells can become follower cells in response to energy depletion during invasive migration, an energy‐demanding process 57. During invasion through matrix barriers, breast cancer cells were found to take up higher levels of glucose, leading to increased energy production. The ATP/ADP ratio of the leader cells was increased in the instant before a major contraction during leader cell invasion. However, mathematical modelling revealed that when this energy drops below a threshold, the leader cells can become followers and a more energetic follower cell will lead until the first leader cell regains its energy. This swapping promoted efficient collective invasion of a cell cluster, whereas delays in this switch between leader/follower cells can disrupt the invasion process 57.

## Pressures driving migration: chemotaxis and self‐generated gradients

Migration of single cells or clusters can be driven and spatially directed by a variety of extracellular chemical cues, a process known as chemotaxis (Figure 3). While chemotaxis towards sources of attractant has been extensively reported and characterised, an emerging concept is that cells can often generate their own chemoattractant gradient by consuming or destroying local supplies. A dramatic example was the discovery that melanoma cells chemotax towards lysophosphatidic acid (LPA), a driver of cancer cell intravasation and metastasis 58. Specifically, primary tumours can rapidly consume LPA, acting as ‘sinks’, eventually motivating cells to migrate out in response to self‐generated gradients 58. This mechanism also works in pancreatic cancer, with LPA promoting metastasis through a signalling loop with the actin modulating protein N‐WASP 59. The LPA receptor, LPAR1, which is thought to drive this chemotactic response, is a G‐protein‐coupled 7‐transmembrane spanning receptor and its endocytic trafficking is directed by N‐WASP 58, 59. Multiple chemokines are also implicated in cancer cell chemotaxis and this has recently been reviewed in 60. Likewise, growth factors such as EGF, PDGF, and HGF are well characterised as stimulators of chemotaxis and are all internalised together with their receptors. Receptor tyrosine kinase signalling is also important in chemotaxis, where Muinonen‐Martin *et al* found that growth factors modulate the speed of translocation of cells in a chemotaxis chamber, but only modestly affect directionality 58. There is also increasing evidence that extracellular vesicles could promote chemotaxis through growth factors and chemokines. Exosomes displaying these cues are able to direct migration (autocrine manner), whereas exosomes that are left behind can be taken up by follower cells enabling directed migration (paracrine manner) 60, 61. Karagiannis *et al* recently reviewed certain exciting efforts to translate our knowledge around chemotaxis of breast cancer cells into signatures that can be used to predict or intervene against metastasis 62. It has emerged from the work of Oktay, Condeelis and co‐workers that haematogenous metastatic dissemination of breast (and other) cancers occurs through a collaboration between macrophages and tumour cells to penetrate blood vessels in a phenomenon called TMEM (tumour microenvironment of metastasis) 63. It is clear that more research into chemotactic drivers and self‐generated gradients will be important to maximally understand how various mediators could be manipulated to better combat tumour spread.

## Pressures driving migration: starvation for nutrients and macropinocytic uptake

Tumours not only consume/destroy attractants rapidly, but they grow and expand faster than they can supply nutrients or oxygen, due to uncontrolled proliferation and poor vascularisation (Figure 3). Cancer cells readily respond to the stress of starvation by ramping up nutrient uptake pathways, utilising alternative sources of energy, and rewiring their metabolism (reviewed in 64, 65). The actin cytoskeleton is highly responsive to these conditions and consequently is a major driving force of nutrient uptake upon energy demand. Furthermore, the starvation response in tumours is linked with cell migration reprogramming in ways that are strikingly conserved in evolution 66. The reprogramming of migration pathways in tumours has been compared with bacterial reprogramming during starvation and yeast pseudohyphal growth induced upon starvation. It involves rewiring of protein translation through the eukaryotic translational initiation factor eIF2a, leading not only to reduced global protein translation but also to a switch of a more invasive and migratory programme of translation and transcription via the ATF4 transcription factor (recently reviewed in 66). Here, we discuss mainly the reprogramming of the cytoskeleton to effect nutrient uptake by macropinocytosis, a method for engulfment of large particles that can serve as a food source.

Excessive activation of Ras or Rac GTPases (such as due to oncogenic mutations) can drive actin cytoskeleton remodelling causing plasma membrane ruffling and bulk engulfment of extracellular material through a process called macropinocytosis 67 (Figure 3). The cell forms cup‐like structures around particles, after which the membranous distal tips fuse together forming a macropinosome. After engulfment, macropinosomes fuse with lysosomes to break down and consume the extracellular material, fuelling survival 68. Macropinocytic cup formation is largely driven by Rac1–Scar/WAVE and Arp2/3 69, 70. However, many aspects of how macropinosomes are formed are unknown. In the early stages, actin organises into a protrusive ring underneath the plasma membrane as a precursor of a macropinocytic cup. A study using super resolution lattice light‐sheet microscopy recently revealed how this ring accumulates active Ras and Rac1 as well as PI3,4,5P_3_ molecules, whereas at the edge of this cup Scar/WAVE is activated. Actin polymerisation at the interface of this nascent cup drives extracellular accumulation and cup closure mediates uptake in gulps 71. Abi, a protein subunit of the Scar/WAVE complex, localises in macropinocytic vesicles during synaptic development in *Drosophila melanogaster*. Upon phosphorylation by Abl kinase, Abi is thought to regulate Rac1 Scar/WAVE‐mediated actin assembly 72. PI3K is also important for macropinosome formation by converting PI4,5P_2_ into PI3,4,5P_3_, which somehow defines regions/boundaries of the macropinosome and enables membrane protrusion extension. This mechanism can be downregulated by the tumour suppressor phosphatase PTEN, which will convert PI3,4,5P_3_ back to PI4,5P_2_ and inhibit the closure of macropinosomes 73.

Macropinocytosis of proteins can fuel pancreatic cancer when other nutrients are in short supply 74. Tumours can break down proteins taken up from the microenvironment by macropinocytosis 75, 76. A recent study has shown that non‐small‐cell lung cancer cells can survive glucose shortage by upregulating Rac1‐mediated macropinocytosis. Macropinocytosed protein can be broken down and used to generate ATP through the activation of the tricarboxylic acid (TCA) cycle, fuelling proliferation 77. PTEN is often inactive or deleted in many cancer types, including prostate cancer, and PTEN‐deficient prostate cancer cells can survive under nutrient starvation by upregulating macropinocytosis through AMPK pathway activation 78. Rather than taking up soluble proteins from the environment (e.g. albumin), PTEN‐deficient prostate cancer cells engulf necrotic cell debris to power protein synthesis and lipid maintenance, processes essential for tumour growth 78. Thus, macropinocytosis can provide tumours with a variety of types of nutrients and may be a process worth targeting in therapy.

Targeting macropinocytosis via the actin cytoskeleton or its regulators could be considered a potential therapeutic target. However, there are no specific inhibitors for macropinocytosis. The Rac1 GEF, DOCK1, is a potential therapeutic target for macropinocytosis in tumours with Rac1^P29S^ mutation, as its inhibition caused a decrease in invasion and macropinocytosis of both melanoma and breast cancer cells harbouring Rac1^P29S^ mutations 79. Moreover, a pharmacological screen recently identified new potential therapeutic agents against macropinocytosis. In this study, about 640 FDA‐approved compounds were screened using various molecular biology techniques and scanning electron microscopy. Imipramine, a tricyclic antidepressant compound, with IC_50_ ≤ 131 nm, was found to inhibit the initial steps of macropinocytic cup formation in various cells including cancer cells 80. Although this compound could be promising, more studies need to be performed to understand the mechanisms of its function as a therapeutic agent in pathologies including cancer.

## Pressures driving migration: cell crowding and unbalanced mechanical forces

Epithelial tissue homeostasis is crucial for maintaining a functional protective barrier and is established through balancing forces between resting, dividing, and dying cells. To allow turnover and prevent overcrowding of cells in an epithelium, dying cells are extruded from the monolayer (Figure 4). In zebrafish epithelium, cell extrusion is achieved by contraction of an actomyosin ring, leading to the excess cells being squeezed out apically to maintain a functional epithelial monolayer 81. Cell extrusion is also a mechanism for elimination of abnormal neighbouring cells, termed EDAC (epithelial defence against cancer). Healthy epithelial cells have the ability to detect cancerous neighbouring cells and apically extrude them from the epithelial tissue (reviewed in 82). This process is also correlated with actomyosin contractility, as Src‐transformed cells that were extruded by normal neighbouring cells showed high levels of myosin‐II 83. Normal epithelial cells can extrude surplus cells apically into the lumen; however, tumours also squeeze cancer cells basally, promoting invasion in response to oncogenic signalling. This aberrant extrusion can allow them to enter the bloodstream and metastasise to distant sites 84 (Figure 4). Extrusion of single cells from epithelia has been highlighted as a way for single cells to disseminate from tumours or even from precancerous sites 81, 84.

Cancer driver mutations, such as Kras^G12V or G12D^, can promote basal cell extrusion, explaining why tumours harbouring such mutations, including pancreatic cancer, can be so invasive and metastatic. The mechanism of extrusion may be via lipid signalling through sphingosine‐1‐phosphate (S1P), as the S1P_2_ receptor is important in the extrusion of pancreatic ductal adenocarcinoma cells driven by the Kras^G12V^ mutation. Deletion of S1P_2_ receptor increased the basal extrusion and increased the invasive properties of cancer cells, whereas re‐introduction of the protein reverted the phenotypes and reduced metastasis 85, 86. Loss of E‐cadherin in mouse mammary epithelial cells can also lead to cell extrusion, but it is not sufficient for formation of invasive lobular carcinomas. The extruded E‐cadherin‐deficient cells showed increased levels of phospho‐myosin light chain and non‐apoptotic membrane blebbing. Reduction of contractility resulted in increased survival and proliferation, as well as the development of invasive lobular carcinoma 87.

Physical pressures derived not only from neighbouring cells but also from the surrounding matrix can trigger cell migration. The structural properties of the ECM and the pericellular microenvironment can influence the directional migration of cancer cells. Cells typically migrate following gradients of increasing stiffness, a substrate stiffness‐mediated directed programme of migration also known as durotaxis. Human cancer cells can also durotax, and they prefer softer stiffness gradients (2–7 kPa), similar to normal soft tissue, whereas upon a threshold of stiffness cells displayed a milder response 88. Inhibition of Arp2/3 complex impaired lamellipodia protrusion and durotaxis 88. In addition to this, neuronal Schwann cells can also migrate directionally and change their phenotype in response to gradients of increasing extracellular stiffness, a response essential for nerve regeneration 89.

Increasing evidence suggests that a migratory cell can sense ECM stiffness through filopodia, finger‐like protrusions generated by actin polymerisation at the leading edge. Filopodia have been implicated in durotactic migration as well as ECM invasion. Filopodia not only sense the rigidity of the environment but they can also promote migration through generation of forces produced by combining actomyosin contractility and binding to ECM fibres 90, 91. Mathematical modelling and experiments in embryonic chick forebrain neurons suggested a stochastic model, the ‘motor‐clutch’ force transmission system, where the retrograde flow of F‐actin in filopodia was faster with less traction forces in matrices with higher stiffness, and the clutch was more engaged and the retrograde flow slower on softer substrata 92. An application for quantification of filopodia microscopically was recently developed and used to show that invading breast cancer cells use filopodia in 3D during invasion 93. Filopodia are also important for invasion of melanoma cells expressing the actin bundling protein fascin‐1 94. However, much still remains unknown about the importance of filopodia in cancer cell force production for matrix remodelling and invasion.

Numerous observations implicate ECM stiffness and mechanical forces in tumour progression, invasion, and metastasis. For example, in breast cancer, stiffening of the ECM and the surrounding stromal tissue can activate mechano‐signalling pathways in cancer cells that will lead to cell proliferation, changes in cell shape, and migration 95. Furthermore, epithelial ovarian cancer cells show enhanced actomyosin contractility in response to greater substrate rigidity 96. This correlated with increased cell spreading, alterations of focal adhesion properties, and enhanced durotaxis 96. Mechanoresponses may be transduced by focal adhesion kinase (FAK), as it is one of the first proteins recruited to focal adhesions in response to an external mechanical stimulus. Upon increased stiffness, active FAK can cause translocation of the transcriptional regulator Yes‐associated protein (YAP) to the nucleus, which can lead to increased expression of genes essential for durotaxis, directed migration, and metastatic spread 97.

The actin cytoskeleton can also drive metastatic spread through generation of forces at the basement membrane. It is widely known that basement membrane breaching occurs in various processes and is important during development as well as immune cell migration and function 98. *Caenorhabditis elegans* provides a fascinating model for normal developmental breaching of basement membrane and invasion during vulval development. A single cell, called the anchor cell, assembles invadopodia and uses both metalloproteases and actin assembly to force its way through the basement membrane. However, it was recently shown that deletion of the genes encoding matrix metalloproteases failed to stop this invasion event. Enhanced F‐actin polymerisation at the protrusive invasive area and recruitment of mitochondria to produce ATP locally at invading sites were also required for anchor cell invasion 99. Thus, local energy production can fuel actin dynamics to generate force‐producing invasive structures. Cancer‐associated fibroblasts (CAFs) also use mechanical forces generated by actin cytoskeleton remodelling to breach the basement membrane and invade through pre‐existing cavities and widen the holes, enabling them to squeeze through and invade 100. Thus, both normal and tumour cells can respond to mechanical barriers by increasing their own force production machinery to overcome barriers and to disseminate.

## Conclusion

Both normal and tumour cells have plastic mechanisms to adapt their migration and invasion strategies according to the various stresses and demands of the environment. Transcriptional regulation of migration programmes is perhaps the best understood among changes driving tumour migration, with a vast literature surrounding the epithelial‐to‐mesenchymal transition. While concepts surrounding epithelial‐to‐mesenchymal transition have made great contributions to our understanding of cancer, we would argue that there is not one programme driving migration changes in tumour cells, but rather an opening up of possibilities due to stress. The cytoskeleton has inbuilt flexibilities to allow cells to cope with normal stresses. Essentially, the cytoskeleton is intimately linked to other cellular systems, such as membrane trafficking, proliferation, energy flow, and polarity. Cancer presents extreme stress to cells and thus they harness flexibilities of normal cytoskeletal programmes which can lead to metastatic spread. If we can better understand the range of responses that cells can make in both normal and diseased states, we can hope to find weaknesses in these strategies and exploit them for better therapies.

## Author contributions statement

LMM and SN wrote and edited the manuscript together. SN drafted and constructed the figures.

**Abbreviations**
CAF: cancer‐associated fibroblast
CLCb: clathrin light chain b
Dyn1: dynamin‐1
EDAC: epithelial defence against cancer
FAK: focal adhesion kinase
LPA: lysophosphatidic acid
PCP: planar cell polarity
RTK: receptor tyrosine kinase
S1P: sphingosine‐1‐phosphate
TMEM: tumour microenvironment of metastasis
